# Identified lncRNAs functional modules and genes in prediabetes with hypertriglyceridemia by weighted gene co-expression network analysis

**DOI:** 10.1186/s12986-022-00665-5

**Published:** 2022-05-02

**Authors:** Mengzi Sun, Shoumeng Yan, Di Zhao, Ling Wang, Tianyu Feng, Yixue Yang, Xiaotong Li, Wenyu Hu, Nan Yao, Weiwei Cui, Bo Li

**Affiliations:** 1 Department of Epidemiology and Biostatistics, School of Public Health, Jilin University, 1163 Xinmin Avenue, Changchun, 130021 People’s Republic of China; 2 Department of Physical Examination Central, The First Hospital of Jilin University, Changchun, 130021 People’s Republic of China; 3 Department of Nutrition and Food Hygiene, School of Public Health, Jilin University, 1163 Xinmin Avenue, Changchun, 130021 People’s Republic of China; 4 Department of Social Medicine and Health Management, School of Public Health, Jilin University, Changchun, 130021 People’s Republic of China

**Keywords:** WGCNA, Prediabetes, Hypertriglyceridemia, lncRNAs, KEGG, PPI

## Abstract

**Background:**

Hypertriglyceridemia (HTG) is one of the most important comorbidities in abnormal glucose patients. The aim of this study was to identify lncRNAs functional modules and hub genes related to triglyceride (TG) in prediabetes.

**Methods:**

The study included 12 prediabetic patients: 6 participants with HTG and 6 participants with normal triglyceride (NTG). Whole peripheral blood RNA sequencing was performed for these samples to establish a lncRNA library. WGCNA, KEGG pathways analysis and the PPI network were used to construct co‐expression network, to obtain modules related to blood glucose, and to detect key lncRNAs. Meanwhile, GEO database and qRT-PCR were used to validate above key lncRNAs.

**Results:**

We found out that the TCONS_00334653 and PVT1, whose target mRNA are MYC and HIST1H2BM, were downregulating in the prediabetes with HTG. Moreover, both of TCONS_00334653 and PVT1 were validated in the GEO database and qRT-PCR.

**Conclusions:**

Therefore, the TCONS_00334653 and PVT1 were detected the key lncRNAs for the prediabetes with HTG, which might be a potential therapeutic or diagnostic target for the treatment of prediabetes with HTG according to the results of validation in the GEO database, qRT-PCR and ROC curves.

**Supplementary Information:**

The online version contains supplementary material available at 10.1186/s12986-022-00665-5.

## Introduction

According to the 2020 National Diabetes Statistics Report from the U.S. Center of Disease Control and Prevention, it was estimated that 34.5% of the adult U.S. population and 46.6% of those aged 65 years and older were prediabetic patients, but only 15.3% of them have known about their prediabetes state [1]. Prediabetic patients will not only develop type 2 diabetes (T2DM), but also have noteworthy risk factors for macrovascular disease [2]. However, the increased risk of cardiovascular events in prediabetic patients might be mediated by lipid abnormalities caused by hyperglycemia [3, 4]. Moreover, atherogenic dyslipidemia (AD) with hypertriglyceridemia (HTG) are the most important comorbidities in T2DM patients, and it was reported that diabetic patients with AD had higher risk of CVD in [5]. It was reported that HTG may be as prevalent as 50% in T2DM [6] and that triglyceride (TG) levels were closely related to insulin resistance-compensated hyperinsulinemia, rather than that simply increased with the increase in hyperglycemia [7]. Therefore, the prevention of HTG in the patients with prediabetes might be significantly important to reduce the risk of CVD.

LncRNAs represent a class of transcripts longer than 200 nucleotides with abilities of DNA-, RNA- and protein-binding [8]. Various studies have reported that their function in the regulation of gene expression, cellular differentiation, and lots of diseases, although the significance of most of lncRNAs has not been identified [9]. It was suggested that lncRNA were involved in the entire prediabetes biological process [10]. For example, lncRNA MALAT1 can regulate renal tubular epithelial pyroptosis by modulating miR-23c targeting of ELAVL1 in T2DM [11]. The plasmacytoma variant translocation 1 (PVT1), a 1.9 kb long lncRNA, is highly expressed in podocytes and mesangial cells after high glucose exposure [12]. It was also suggested that PVT1 to be dramatically upregulated in mice with streptozotocin (STZ)-induced diabetes [13]. However, conventional method usually described the correlation structure between thousands of genes and a sample trait [14]. Fortunately, weighted gene co-expression network analysis (WGCNA) could solve the problem.

WGCNA is used to explore the clusters (modules) of highly correlated genes, to summarize such clusters using the module eigengene or an intramodular hub gene. WGCNA could also relate modules to one another and to external sample traits (using eigengene network methodology), and calculate module membership measures [15]. In the WGCNA algorithm, the elements in the co‑expression matrix of the genes were no longer the correlation coefficients of the genes, but rather the weighted value of the correlation coefficients [16]. Based on the above advantages of WGCNA, we aimed to identify lncRNAs functional modules and hub genes related to TG in prediabetes, which to find a potential therapeutic or diagnostic target for the treatment of prediabetes with HTG.

## Materials and methods

### Participants

This study involved 12 patients with prediabetes, 6 participants were diagnosed as HTG and the other 6 participants had normal TG level (NTG). All participants were Chinese aged 40–65 years, which were recruited at the First Hospital of Jilin University from July to September 2020. Patients who have used drugs or other treatments to control blood glucose or TG in the past, or have a history of coronary artery disease (CAD), hypertension, atrial fibrillation, myocardial infarction, tumor, acute infectious disease, immune disease, hematological disease were excluded in our study. All participants have written informed consent and the study was approved by Ethics Committee of the Public Health of the Jilin University, and the privacy of the participants are strictly confidential.

The diagnostic criteria of prediabetes and HTG were based on the “Guidelines for the Prevention and Control of Type 2 Diabetes in China” (2017 Edition) and the “Guidelines for Prevention and Treatment of Dyslipidemia in Adults in China” (2016 Edition). Patients with prediabetes were defined as whose fasting blood glucose (FBG) ranging from 6.1 to 7.0 mmol/L or oral glucose tolerance test (OGTT) two-hour blood glucose ranging from 7.8 to 11.1 mmol/L. Patients with TG > 1.7 mmol/L were defined as HTG.

### Blood sample collection and RNA sequencing

Trizol (TAKARA BIO INC., CA, Japan) was added immediately after the blood samples were collected. Total RNA extraction kit was used to isolate and purify the total RNA. The RNA purity was tested using NanoPhotometer® spectrophotometer (IMPLEN, CA, USA) and the RNA integrity was evaluated using RNA Nano 6000 Assay Kit of the Agilent Bioanalyzer 2100 system (Agilent Technologies, CA, USA).

The chain-specific library was constructed with ribosomal RNA removing, and was sequenced according to pooling of the effective concentration of the library and the data output requirements, which using the Illumina PE150. The reads with adapter, with the nucleobase information cannot be determined (N) ≥ 0.002, and with low-quality from raw data were removed for followed sequencing with calculating Q20, Q30, and GC content additionally. All analyses in the study were based on the clean data obtained through the above criteria.

### Construction of WGCNA

The “WGCNA” [17] package in R-Studio 4.0.4 software was used for data analysis, which is a comprehensive collection of R functions for performing various aspects of weighted correlation network analysis [18]. WGCNA analysis focuses on the association between the sample trait and a few modules, instead of describing the correlation structure between thousands of genes and a sample trait [14]. In the WGCNA algorithm, the elements in the co‑expression matrix of the genes are no longer the correlation coefficients of the genes, but rather the weighted value of the correlation coefficients [16]. The lncRNAs whose Median Absolute Deviation (MAD) > 0.01 were selected for the subsequent analysis to ensure heterogeneity and accuracy of bioinformatics for co‐ expression network analysis. Pearson correlation coefficient were calculated for all the genes, and an appropriate soft threshold *β* was automatically selected through the pickSoft-Threshold function in the WGCNA package whose function was to amplify the correlation between genes [19], it was 0.85 in this study.

Finally, a dynamic tree was used to divide the modules of hierarchical clustering results, and merge the modules with lncRNAs < 30 and cutting height < 0.25 [20].

### Screening for key modules

Based on the above analysis, we subdivided nearly two thousand genes into several modules. Modules were defined as a set of genes in which the expression mode highly correlated with the sample and the first principal component module characteristic genes (MEs) were calculated to express the expression level of the gene module. The strongest correlation module with prediabetic HTG was determined whose absolute value of Pearson's correlation coefficient > 0.8 and *P-*value < 0.1 [21] in this study.

### Identification of key lncRNAs and functional enrichment analysis

We put the genes of the strongest correlation module whose Module membership (MM) > 0.5 and gene significance (GS) > 0.2 into the representative Kyoto Encyclopedia of Genes and Genomes (KEGG) pathways analysis for further elucidation of the functional properties. The Search Tool for the Retrieval of Interacting Genes (STRING; Szklarczyk et al.) was used to dissect the protein–protein interaction (PPI) network. In this study, we calculated the degree of genes by network analyzer (a tool in Cytoscape software (https://cytoscape.org/)). Genes with degree ranked top 10 were selected to be hub genes in the PPI network.

### Validation in the GEO data set

GSE130991, is a previously published GEO data which data and sample collection took place in France between 2006 and 2016 [22]. There were 97 prediabetic patients who met criteria of our study aim, 28 of them with HTG and 69 of them with normal TG level. Data for GSE130991 were obtained by GPL20265 (HTA-2_0) Affymetrix Human Transcriptome Array 2.0. Data were analyzed in Partek Genomics Suite 6.6, normalized using RMA, and log2 transformed. Based on this database, differential expression analyses of genes in GSE130991 were performed using a *t*-test.

Individual *p *values and log2 values (fold change) were obtained. Then, the expression change of selected lncRNAs between prediabetic patients with HTG and NTG groups in the RNA sequencing results was validated by GSE130991.

### Quantitative real‐time polymerase chain reaction (qRT‐PCR) and ROC curves of relative expression of lncRNAs and HTG

The blood samples in qRT-PCR experiment were collected at the First Hospital of Jilin University from July to September 2020 and July 5th to 19th 2021, including participants from previous RNA sequencing. There were 99 prediabetes patients with HTG and 98 prediabetes patients with NTG who met the inclusion criteria, respectively. The total RNA was extracted using the MolPure® Blood RNA Kit (19241ES50, YEASEN) based on the manufacturer’s instructions. Subsequently, we used lnRcute lncRNA First-Strand cDNA Kit (KR202, TIANGEN) to conduct reverse transcription. The cDNA was then analyzed by qRT-PCR using lnRcute lncRNA qPCR Kit (FP402, TIANGEN) on QuantStudio 3 system (Applied Biosystems). The PCR amplification was performed with one cycle at 95 °C for 3 min, followed by 40 cycles at 95 °C for 5 s, at 55 °C for 10 s, and at 72 °C for 15 s. The following PCR primers were used: TCONS_00334653 primers, forward: 5′- AGGAGTTGGAGACAGCGACTAGAG -3′, reverse: 5′- CGTGATGCTTGTTTGCCCAGTTTC -3′; PVT1 primers, forward: 5′- GCTGTGGCTGAATGCCTCAT -3′, reverse: 5′- TCTCAACCCTCTCAGCCAGC -3′. Expression data were normalized to the expression of β-actin with the 2^−ΔΔCt^ method.

Pearson correlation analysis was used to determine the correlation between the relative expressions of lncRNAs and HTG, the significance was set as *P* < 0.05. Roc curves were used to explore the diagnostic efficacy of the relative expression level for HTG.

## Results

### WGCNA constructions

**Fig. 1:**
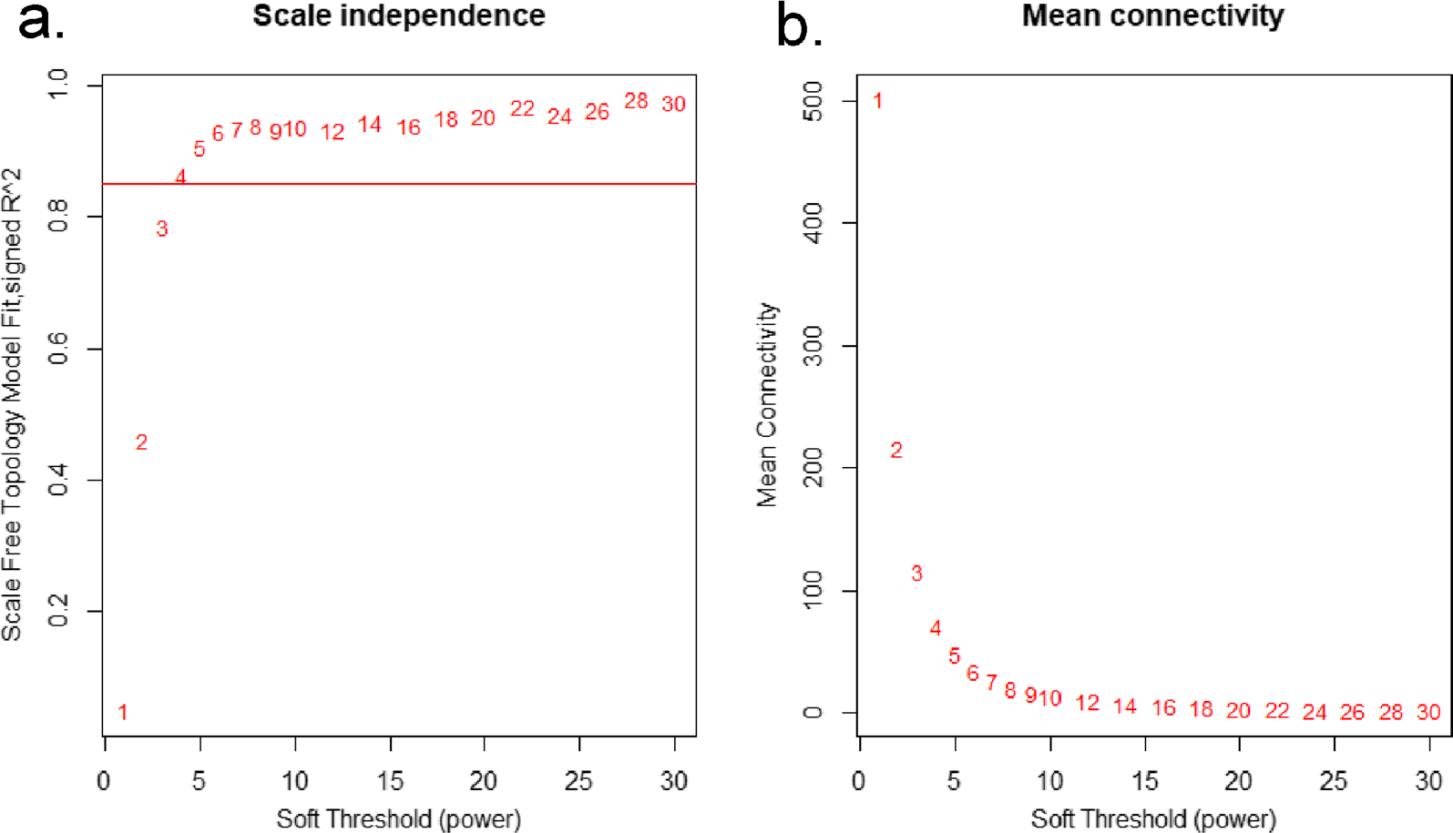
Analysis of the scale-free fit index for various soft-thresholding powers. **a** The chart showed the correlation coefficients of log(k) and log(p(k)) corresponding to different soft thresholds; **b** the chart showed the mean values of gene adjacency coefficients corresponding to different soft thresholds, reflecting the average connectivity level of the network

**Fig. 2:**
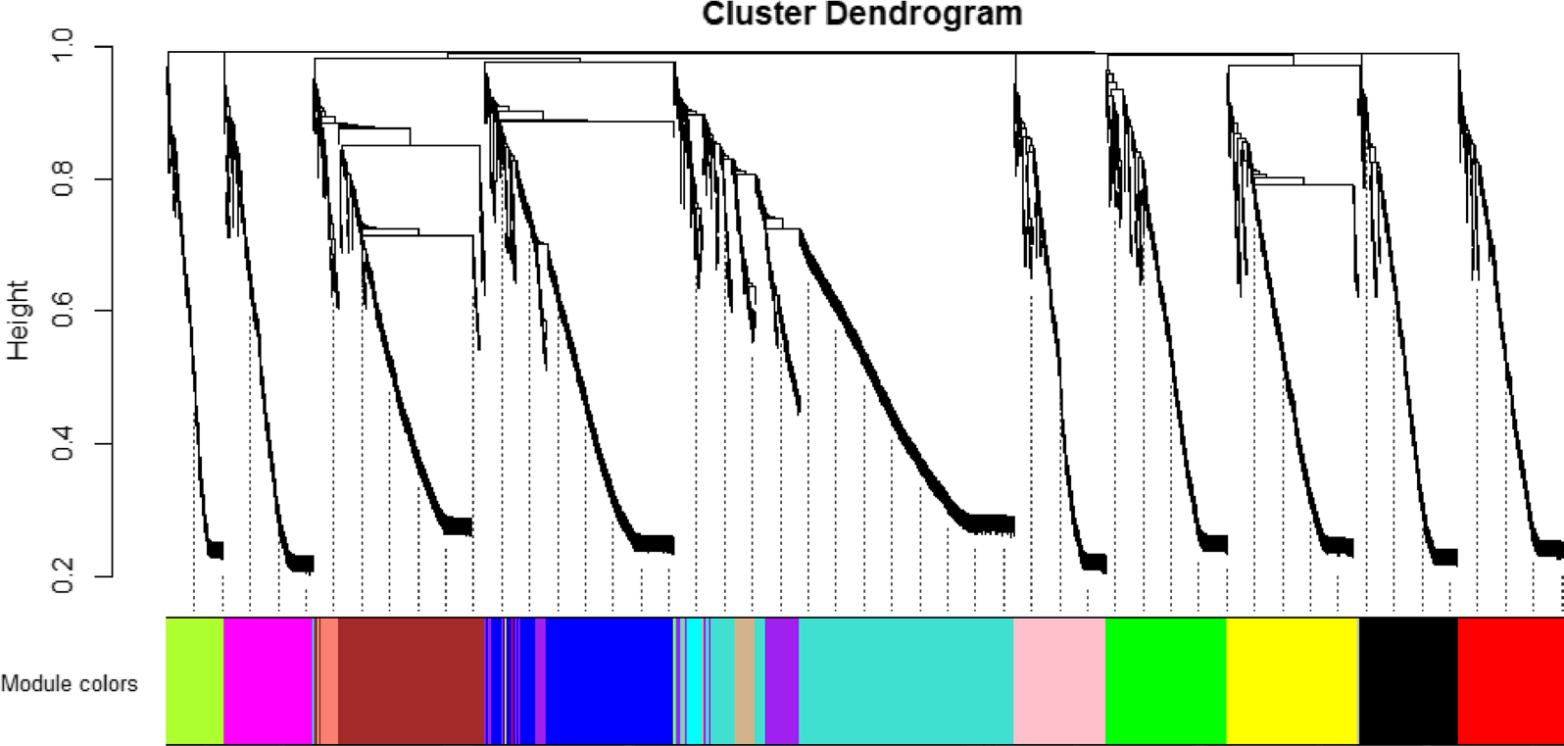
Hierarchical clustering tree and co-expression module of lncRNAs. At the top of the graph was a clustering tree of lncRNAs, and at the bottom were different modules cut from the dynamic cutting tree (different colors represent different modules)

1742 lncRNAs were involved in subsequent analysis after screening all of them based on the mad value greater than 0.01 among total of 7324 lncRNAs. As shown in Fig. 1a, b, the scale-free topology index was 0.85 when the soft-threshold power was defined as 4, which the network conformed to the power-law distribution and closer to the real biological network state. The dynamic hierarchical tree cutting algorithm was used to detect co-expression module according to the weight of lncRNAs, and the results of modules were shown as Fig. 2. We have merged the modules with the number of lncRNAs was less than 30 and the height of the merged module was set to 0.25.

Finally, black, blue, brown, green, greenyellow, grey, magenta, pink, purple, red, salmon, tan, turquoise, yellow (different colors represent different modules) 14 modules were obtained, and the number of every modules were shown in Additional file 1: Table S1.

### Identifying key clinically significant modules

**Fig. 3:**
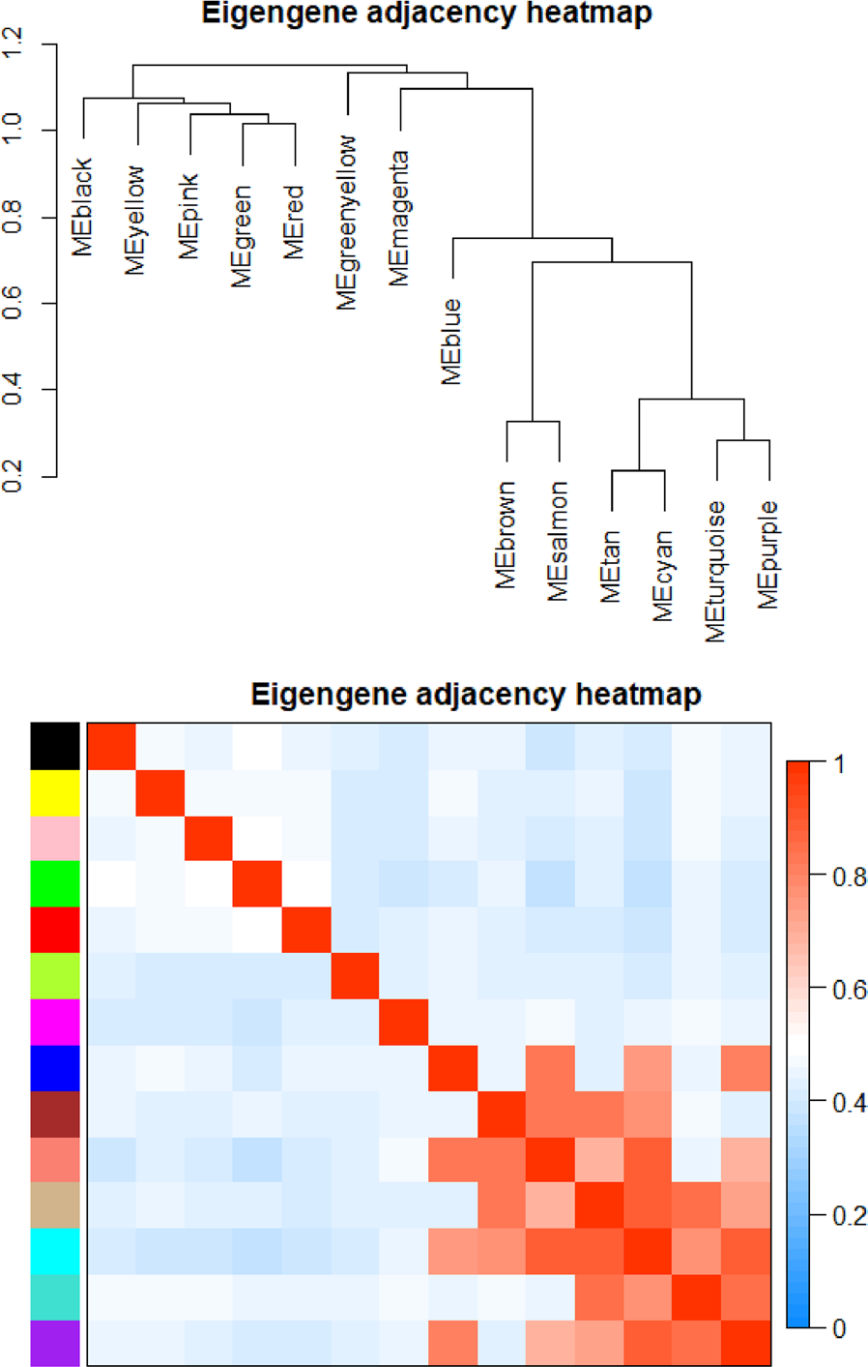
The heat map plot of the adjacencies of modules (Red represented high adjacency (positive correlation), while blue color represented low adjacency (negative correlation))

**Fig. 4:**
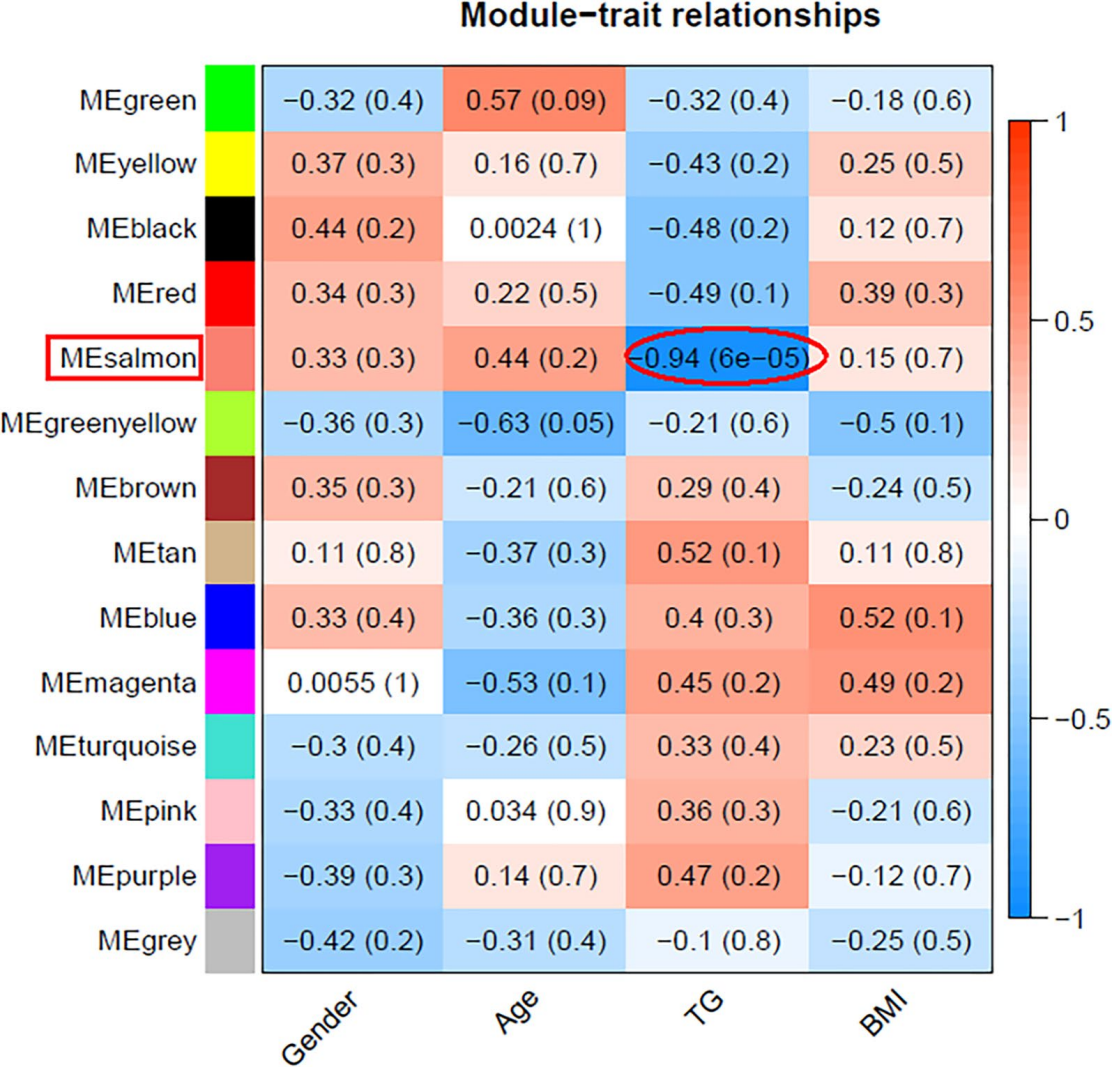
Heatmap of the module–trait relationships. It was represented the Pearson correlation coefficients and P-values of the correlation. Each row corresponded to a module gene, column to a trait. The cells were color coded by correlation according to the color legend

Figure 3 was the heat map plot of the adjacencies of modules which represented the correlation between different modules. The most representative gene set in each module represented the overall level of gene expression in the module as the first principal component of the module eigengene (ME). Two modules corresponding to the sample trait were finally extracted for further functional enrichment analysis, and it was the salmon module had the strongest negative correlation with TG in 6 samples in this study (correlation coefficient = − 0.94, *P* < 0.001) (Fig. 4).

### Hub gene identification and functional annotation

**Fig. 5:**
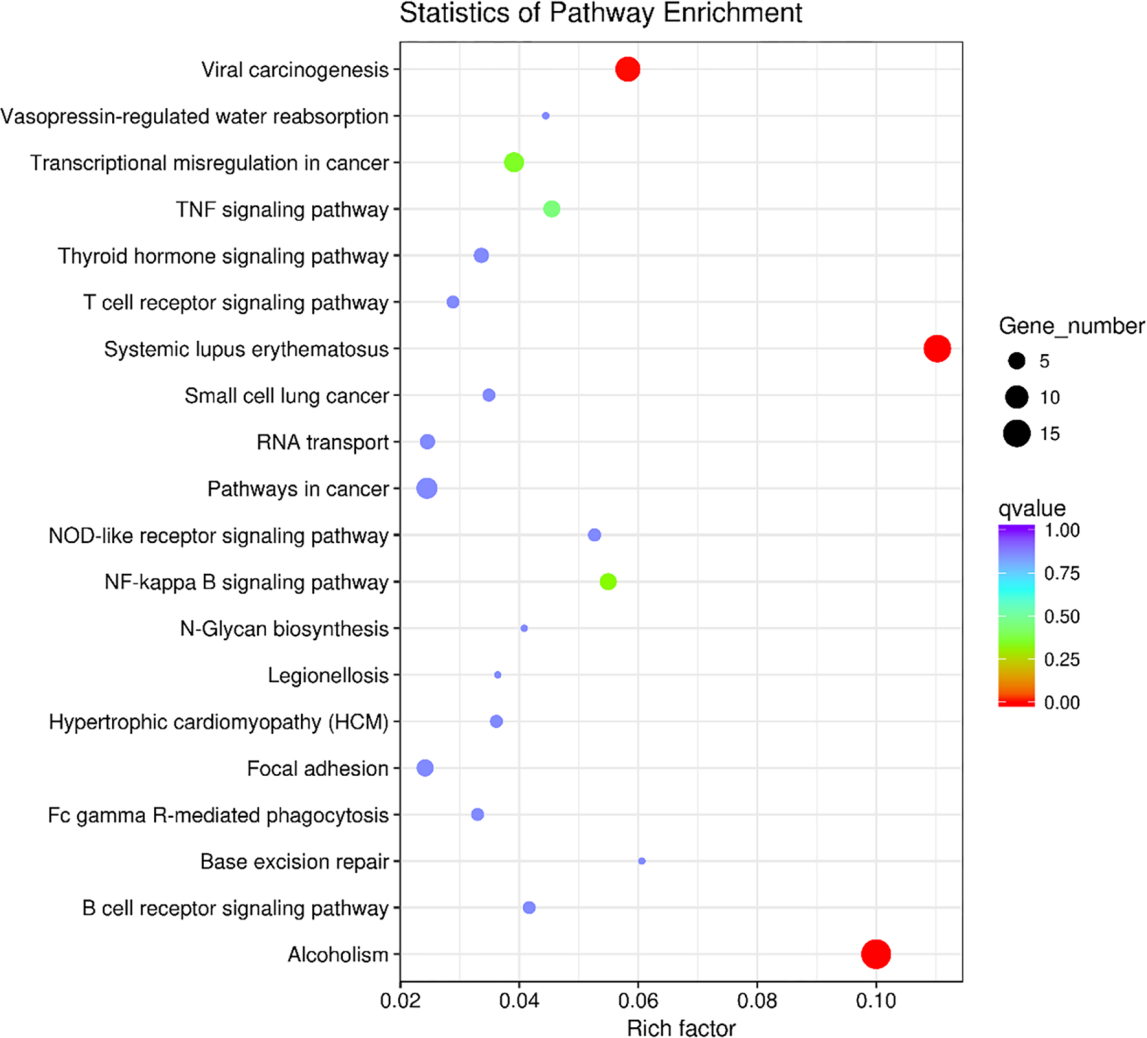
Top 20KEGG pathways of lncRNAs in prediabetes with HTG. The X-axis shows the rich factor and the Y-axis showed the KEGG terms

We screened out lncRNAs in the salmon module according to MM > 0.5 and GS > 0.2, 374 target genes were finally selected for subsequent analysis. KEGG pathways for further elucidation of the functional properties, and it was shown in Fig. 5. The top 7 pathways were significantly different in the results of KEGG, which were Alcoholism, Systemic lupus erythematosus (SLE), Viral carcinogenesis, NF-kappa B signaling pathway, Transcriptional misregulation in cancer, TNF signaling pathway and NOD-like receptor signaling pathway, as shown in Additional file 1: Table S2.

### PPI network

The STRING database was used to dissect the PPI network. There were 216 nodes and 450 edges in the PPI network, which represented proteins and interactions. The expected number of edges was 233 and the *P-*value of PPI enrichment was less than 0.001.

**Fig. 6:**
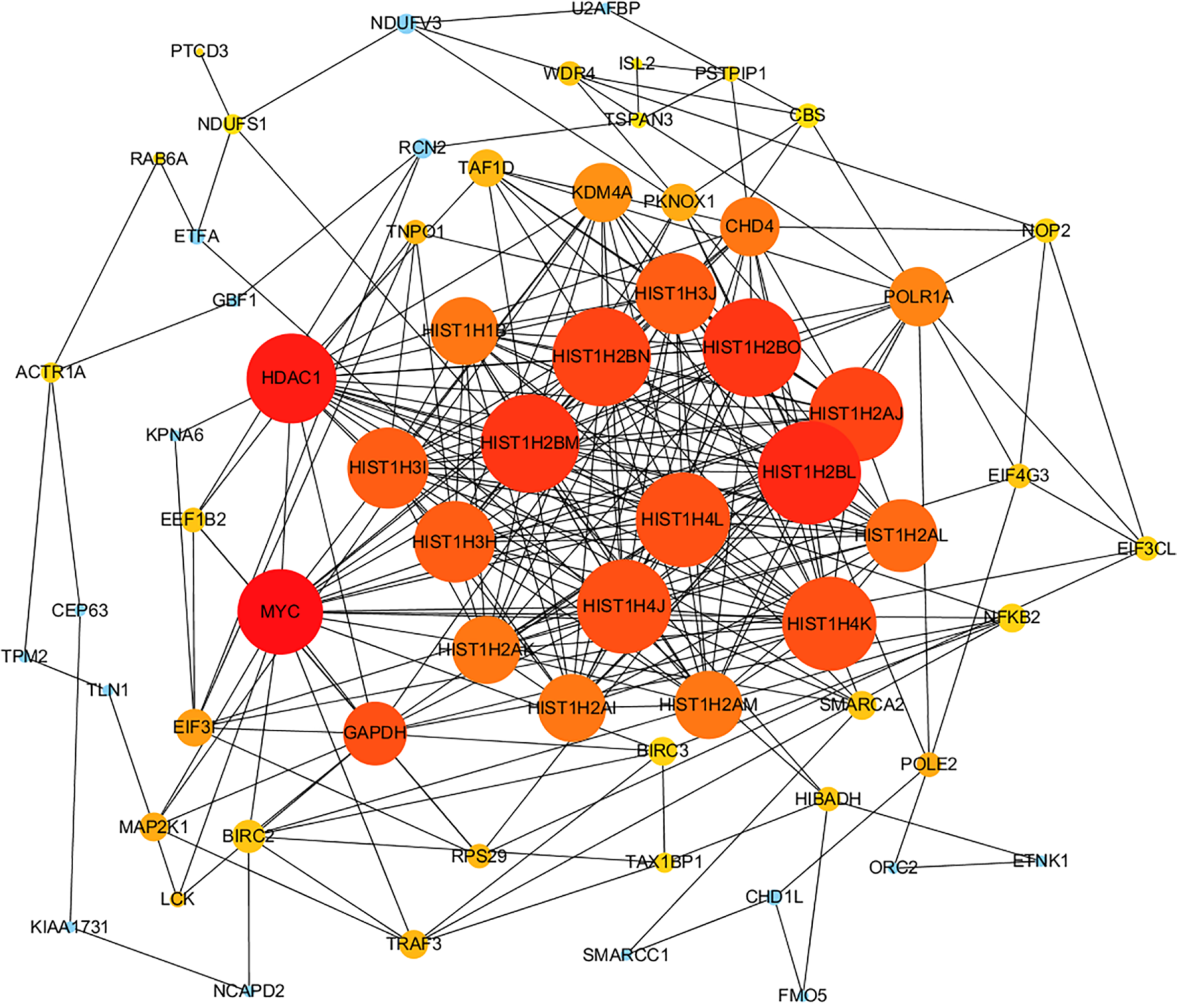
The PPI analysis of target gene of hub lncRNAs. Edge stood for the interaction between two genes. A degree was used for describing the importance of protein nodes (red represented high degree and blue represented low degree). (For interpretation of the references to color in this figure legend, the reader is referred to the web version of this article.)

A visual PPI network based on target genes was constructed using Cytoscape software, which confidence score > 0.4 was set as significant, and the top 11 proteins of connectivity were obtained by cytohubba plug-in. The results of PPI network were shown in Fig. 6 and the degree of top 11 proteins was shown in Additional file 1: Fig. S1. In PPI networks, which the depth of color represented the strength of the connection, “hubs” was regarded as proteins with highly strength of the connection with several other proteins. In this study, the nodes with the higher degree were selected as central proteins that may contribute to prediabetes with HTG in PPI networks.

### Validation in the GEO data set and qRT-PCR

The expression patterns of the top 11 genes were verified by the GSE130991 data set. RNA sequencing is superior to microarray for characterizing transcriptomes, however, data for GSE130991 were obtained by GPL20265 (HTA-2_0) Affymetrix Human Transcriptome Array 2.0. The probes in the data set are early and not enough to detect all genes. Two hub genes of mRNA (MYC and HIST1H2BM, the corresponding lncRNAs of them are PVT1 and TCONS_00334653) met the differential expression criteria of *p* < 0.05 (Additional file 1: Table S3). The HIST1H2BM was also corresponding to the top 3 pathways in the results of KEGG.

**Fig. 7:**
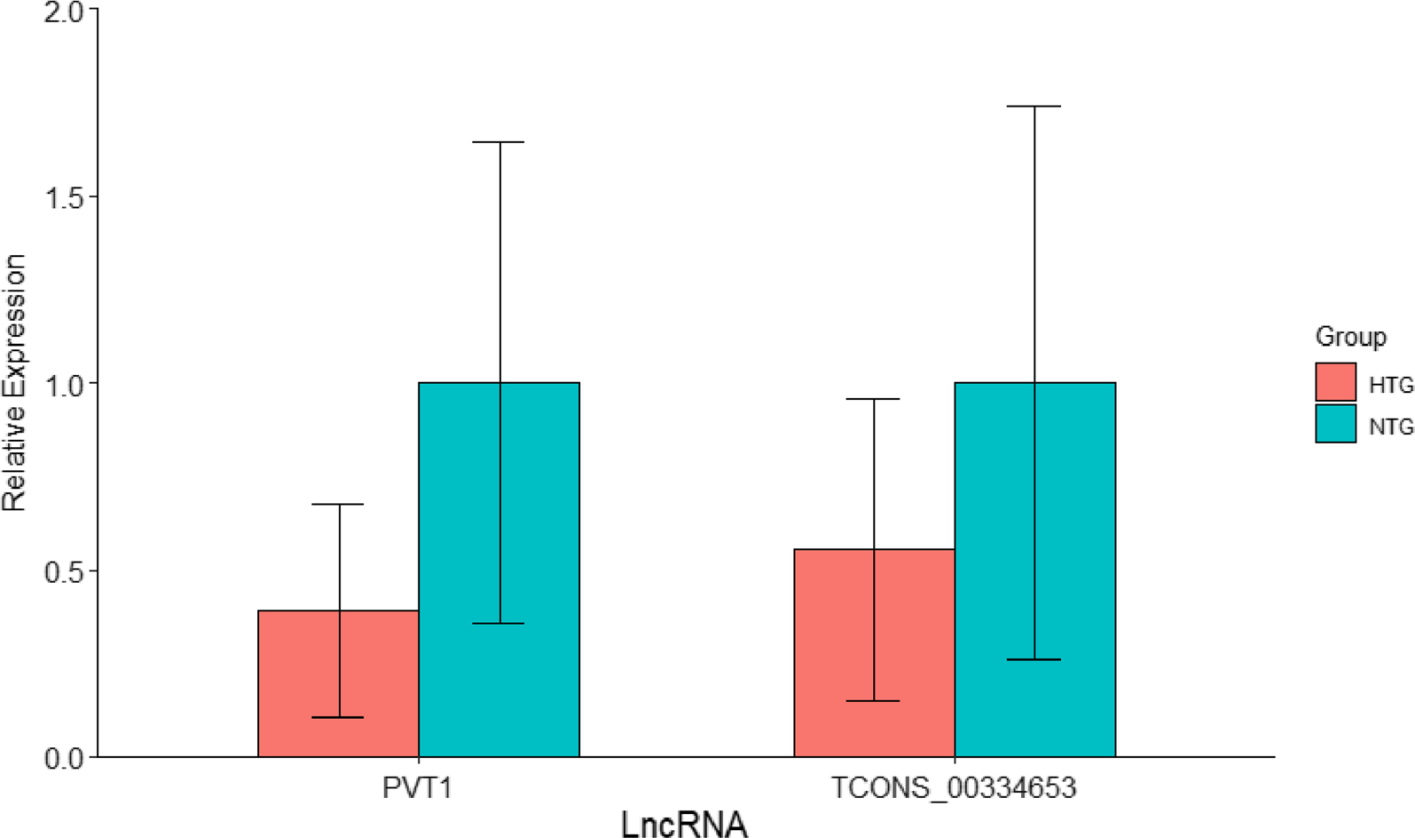
The relative expression and its standard deviation of lncRNA (PVT1 and TCONS_00334653) in the prediabetes with HTG and NTG, respectively

Therefore, we selected the PVT1 and TCONS_00334653 as final lncRNA to validate by the qRT-PCR. As shown in Fig. 7, there were significant difference between prediabetes with HTG and NTG, both of PVT1 (z = 40.400, *P* < 0.001) and TCONS_00334653 (z = 5.757, *P* = 0.016).

**Fig. 8:**
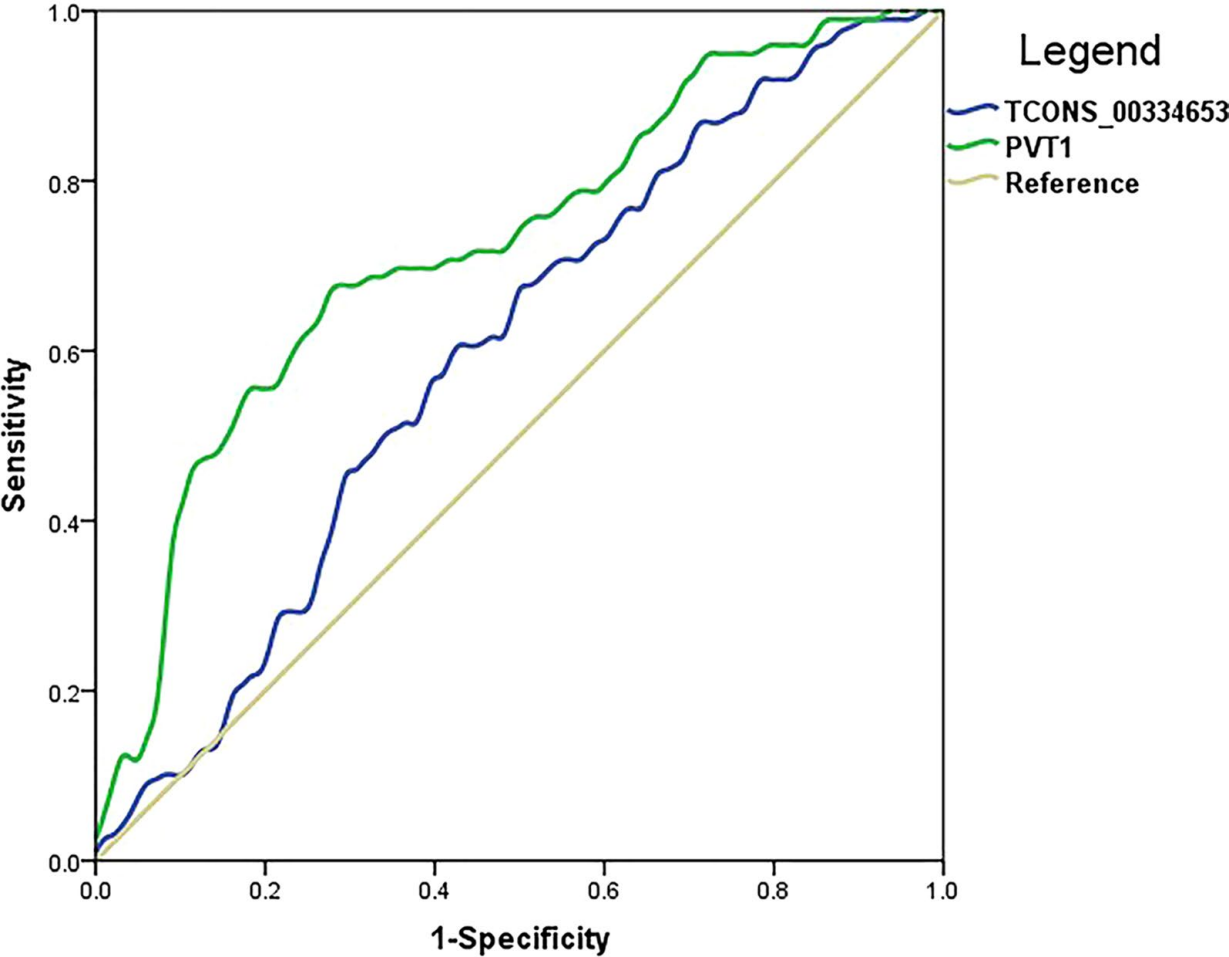
The ROC curves for the relative expressions of lncRNAs PVT1 (green) and TCONS_00334653 (blue) in the prediabetes with HTG

Moreover, the correlations between relative expressions of lncRNAs and HTG were statistically significant (PVT1: correlation coefficient = − 0.366, *P* < 0.001; TCONS_00334653: correlation coefficient = − 0.212, *P* = 0.003). The ROC curves for the relative expressions of lncRNAs PVT1(AUC = 0.724, 95%CI 0.653–0.795, *P* < 0.001) and TCONS_00334653 (AUC = 0.599, 95%CI 0.520–0.678, *P* = 0.016) in the prediabetes with HTG were shown in the Fig. 8. These curves and corresponding AUCs showed that lncRNAs PVT1 and TCONS_00334653 as biomarkers have diagnosed ability to discriminate HTG from NTG in the prediabetes patients.

## Discussion

Dyslipidemia in T2DM is very common and is characterized by HTG with decreased levels of high-density lipoprotein (HDL)-cholesterol [23]. It is important to find a potential therapeutic or diagnostic target for the treatment of prediabetes with HTG, and the main finding of our study are as following. Firstly, top 4 pathways (Alcoholism, SLE, Viral carcinogenesis, and NF-kappa B signaling pathway) might be functionally significant. Secondly, two hub genes of mRNA were HIST1H2BM and MYC, and the corresponding lncRNAs of them are TCONS_00334653 and PVT1. Lipid metabolism is complex due to that included de novo biosynthesis and oxidative catabolism, which products and intermediates within numerous metabolic pathways could contribute to lipogenesis [24].

TCONS_00334653, one of the different lncRNA between prediabetic patients with HTG and NTG, which the target mRNA is HIST1H2BM, was corresponding to the pathways of Alcoholism, SLE and Viral carcinogenesis. Previous study has indicated that damage occurred to the lipid metabolic function in the animal models of ethanol-induced liver-injury, which was manifested by increased levels of TC and TG [25]. Moreover, alcohol emits toxicity when it is just ingested, and the alcohol metabolite acetaldehyde is highly toxic in the body, which can affect multiple organs and cause physiological effects, resulting in various metabolic diseases [26]. Previous studies suggested that alcohol use is a risk factor for the development of IR [27, 28] and the toxic effect of alcohol on pancreatic B cells has been shown to contribute to the development of T2DM [29]. Besides, it is the dose-dependent effect of alcohol consumption in the development of prediabetes [30]. TG level was found as an independent risk factor for i-IGT among men and resulting in a 23.4% increase in the prevalence of i-IGT with each 1-mmol/L increase in TG level [31].

It was reported that the prevalence rate of diabetes, dyslipidemia, elevated TG was found significantly higher in a retrospective study in lupus patients than those in healthy population [32]. The impaired activity of lipoprotein lipase (LPL) in SLE patients, which resulting in accumulation of chylomicrons and very low-density lipoprotein (VLDL), while increased TG and decreased HDL levels [33]. The chylomicron TG could be split off by LPL, using apolipoprotein CII (Apo-CII) allowing the delivery of free fatty acids to adipose tissue and muscle [34]. It was indicated that chylomicron transport is supposed to play a crucial role in the “lupus pattern” of dyslipoproteinemia [35]. Previous studies have found that viral carcinogenesis was associated with glucose and lipid metabolism. For example, Epstein-Barr virus (EBV) is a gamma herpesvirus that is highly prevalent in the human population, which almost all adults are seropositive [36]. It was reported that the prevalence of EBV was significantly higher in diabetic patients than in the individuals without diabetes [37]. EBV has been indicated to manipulate host cell lipid metabolism in both epithelial and B cells, and it was indicated that manipulation of lipid metabolism may play a role in host cell transformation and carcinogenesis [36]. Therefore, the mRNA HIST1H2BM in the above pathways might play a role in participating in lipid metabolism. Moreover, the downregulating protein HIST1H2BM was also found that may be involved in regulating lipid metabolism or other signaling pathways in the acute phase of spinal cord injury [38].

MYC could encode a transcription factor, which is delicately regulated due to its central role in cell proliferation and apoptosis [39]. Previous studies suggested that the MYC oncogene is often activated and/or overexpressed in cancers [40, 41]. It was demonstrated that MYC regulates virtually all stages of lipogenesis, which is required for the initiation and maintenance of tumor growth [42]. And other oncogenes depending on FA synthesis appear to have higher sensitivity to inhibition of lipogenesis when MYC overexpressed [43]. Moreover, in cancer, although MYC overexpression maximizes unrestrained growth, it is vulnerability to the inhibition of lipogenesis [42]. As for PVT1, the corresponding lncRNA of MYC, although it was confirmed to be associated with a variety of malignancies and promote tumor cell proliferation, migration, tumor growth and metastasis [44, 45], and adipogenic potential [46], the lipid metabolism of PVT1 regulating is still unclear. Previous studies indicated that upregulated PVT1 could lead to the damage of biosynthesis [13]. However, our study found out PVT1 was downregulating in the prediabetes with HTG, it might be consistent with that overexpressed MYC is vulnerability to the inhibition of lipogenesis [42]. Besides, it was also found that the PVT1 expression was lower in the gestational diabetes mellitus and preeclampsia placentas than normal placentas [47]. Therefore, downregulated PVT1 could also cause to damage in some extent and it might play a role for the lipid metabolism in the prediabetes.

We also found the correlation between lncRNAs TCONS_00334653 and PVT1 were statistically significant. Therefore, based on the results of present study, we suggested that lncRNAs TCONS_00334653 and PVT1 might be the potential therapeutic or diagnostic target for the treatment of prediabetes with HTG. However, our study has some limitations. In order to obtain reliable information, the results need to be extended to a larger population for exploration. Besides, more molecular biology experiments and functional studies are required to explore the mechanism by the key lncRNAs regulate lipid metabolism in the prediabetes.

## Conclusions

The TCONS_00334653 and PVT1 were detected the key lncRNAs for the prediabetes with HTG, which might be a potential therapeutic or diagnostic target for the treatment of prediabetes with HTG.

## Supplementary Information

**Additional file 1:** Supplementary Materials.

## Data Availability

The datasets for the study are available from the corresponding author on a reasonable request.

## Abbreviations

HTG: Hypertriglyceridemia
KEGG: Kyoto encyclopedia of genes and genomes
LPL: Lipoprotein lipase
MAD: Median absolute deviation
MEs: Module characteristic genes
MM: Module membership
NTG: Normal triglyceride
OGTT: Oral glucose tolerance test
PPI: Protein–protein interaction
PVT1: Plasmacytoma variant translocation 1
SLE: Systemic lupus erythematosus
STRING: Search Tool for the Retrieval of Interacting Genes
STZ: Streptozotocin
T2DM: Type 2 diabetes
TG: Triglyceride
VLDL: Very low-density lipoprotein
WGCNA: Weighted gene co-expression network analysis
