# Supplementary material for: Identified lncRNAs functional modules and genes in prediabetes with hypertriglyceridemia by weighted gene co-expression network analysis
Source: Nutr Metab (Lond). 2022 May 2;19:33. doi: 10.1186/s12986-022-00665-5 (PMC9063339; doi:10.1186/s12986-022-00665-5)
Supplement: Supplementary file 1 — Additional file 1: Supplementary Materials. [file 12986_2022_665_MOESM1_ESM.docx]

**Supplementary materials**

**Identified lncRNAs functional modules and genes in Prediabetes with Hypertriglyceridemia by Weighted Gene Co-expression Network Analysis**

Table S1. Number of lncRNAs contained in different modules

| Module | Number |  | Module | Number |
| --- | --- | --- | --- | --- |
| Black | 118 |  | Pink | 99 |
| Blue | 232 |  | Purple | 81 |
| Brown | 199 |  | Red | 120 |
| Green | 124 |  | Salmon | 53 |
| Greenyellow | 80 |  | Tan | 79 |
| Grey | 20 |  | Turquoise | 323 |
| Magenta | 84 |  | Yellow | 130 |

Table S2. Genes corresponding to significant different pathways by KEGG

| Pathway | Genes | P-value |
| --- | --- | --- |
| Alcoholism | HIST1H3J, HIST1H2AK, HDAC1, HIST1H2AI, CREB3, HIST1H2BN, HIST1H3H, HIST1H4K, HIST1H3I, HIST1H4L, HIST1H2BM, HIST1H2AL, HIST1H4J, HIST1H2BO, HIST1H2AJ, MAP2K1, HIST1H2BL, HIST1H2AM | <0.001 |
| Systemic lupus erythematosus | HIST1H3J, HIST1H2AK, HIST1H2AI, HIST1H3H, HIST1H2BN, HIST1H4K, HIST1H3I, HIST1H4L, HIST1H2BM, HIST1H4J, HIST1H2BO, HIST1H2AJ, HIST1H2AL, HIST1H2BL, HIST1H2AM | <0.001 |
| Viral carcinogenesis | HIST1H2BO, CREB3, TRAF3, HIST1H2BN, HIST1H4K, CHD4, HIST1H4L, HIST1H2BM, HIST1H2BL, HIST1H4J, HDAC1, NFKB2 | <0.001 |
| NF-kappa B signaling pathway | BIRC3, TRAF3, BIRC2, NFKB2, LCK | 0.009 |
| Transcriptional misregulation in cancer | BIRC3, HIST1H3J, ZEB1, NUPR1, HIST1H3I, HIST1H3H, HDAC1 | 0.012 |
| TNF signaling pathway | BIRC3, CREB3, TRAF3, BIRC2, MAP2K1 | 0.018 |
| NOD-like receptor signaling pathway | BIRC3, PSTPIP1, BIRC2 | 0.044 |

Table S3. Hub genes validated in the GSE130991

| Rank | Name | *P-*value | Log2FoldChange |
| --- | --- | --- | --- |
| 1 | **MYC** | 0.0212984 | -0.105 |
| 2 | HDAC1 | 0.1626976 | 0.0367 |
| 3 | HIST1H2BL | 0.9680966 | 0.00214 |
| 4 | **HIST1H2BM** | 0.0133764 | -0.148 |
| 4 | HIST1H2BO | 0.4308774 | -0.0389 |
| 6 | HIST1H2AJ | 0.1230534 | -0.0635 |
| 6 | HIST1H2BN | 0.3726903 | -0.0459 |
| 8 | GAPDH | 0.2890148 | 0.0696 |
| 8 | HIST1H4J | 0.8517497 | 0.0098 |
| 8 | HIST1H4K | -- |  |


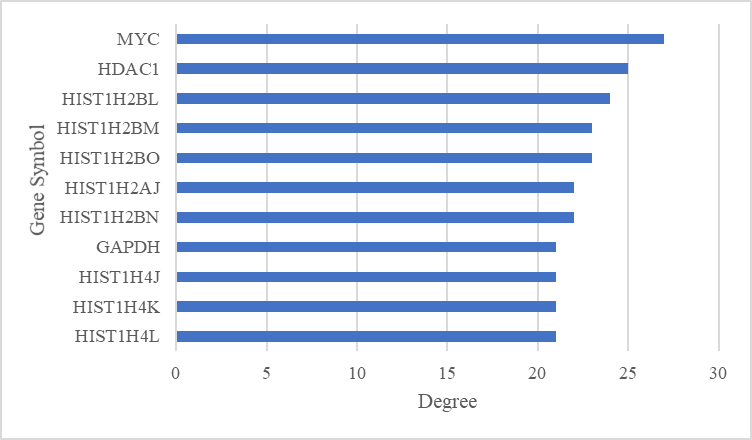


Fig. S1 The degree of the top 11 protein nodes.
